# Supplementary material for: Integrated ‘omics analysis reveals human milk oligosaccharide biosynthesis programs in human lactocytes
Source: iScience. 2025 Aug 7;28(9):113269. doi: 10.1016/j.isci.2025.113269 (PMC12396498; doi:10.1016/j.isci.2025.113269)
Supplement: Document S1. Figures S1–S5 [file mmc1.pdf]

iScience, Volume *28*

Supplemental information

Integrated `omics analysis reveals human milk oligosaccharide biosynthesis programs in human lactocytes

Sarah Kate Nyquist, Laasya Devi Annepureddy, Kristija Sejane, Annalee Furst, G. Devon Trahan, Michael C. Rudolph, Alecia-Jane Twigger, Lars Bode, Barbara E. Engelhardt, Jayne F. Martin Carli, and Britt Anne Goods

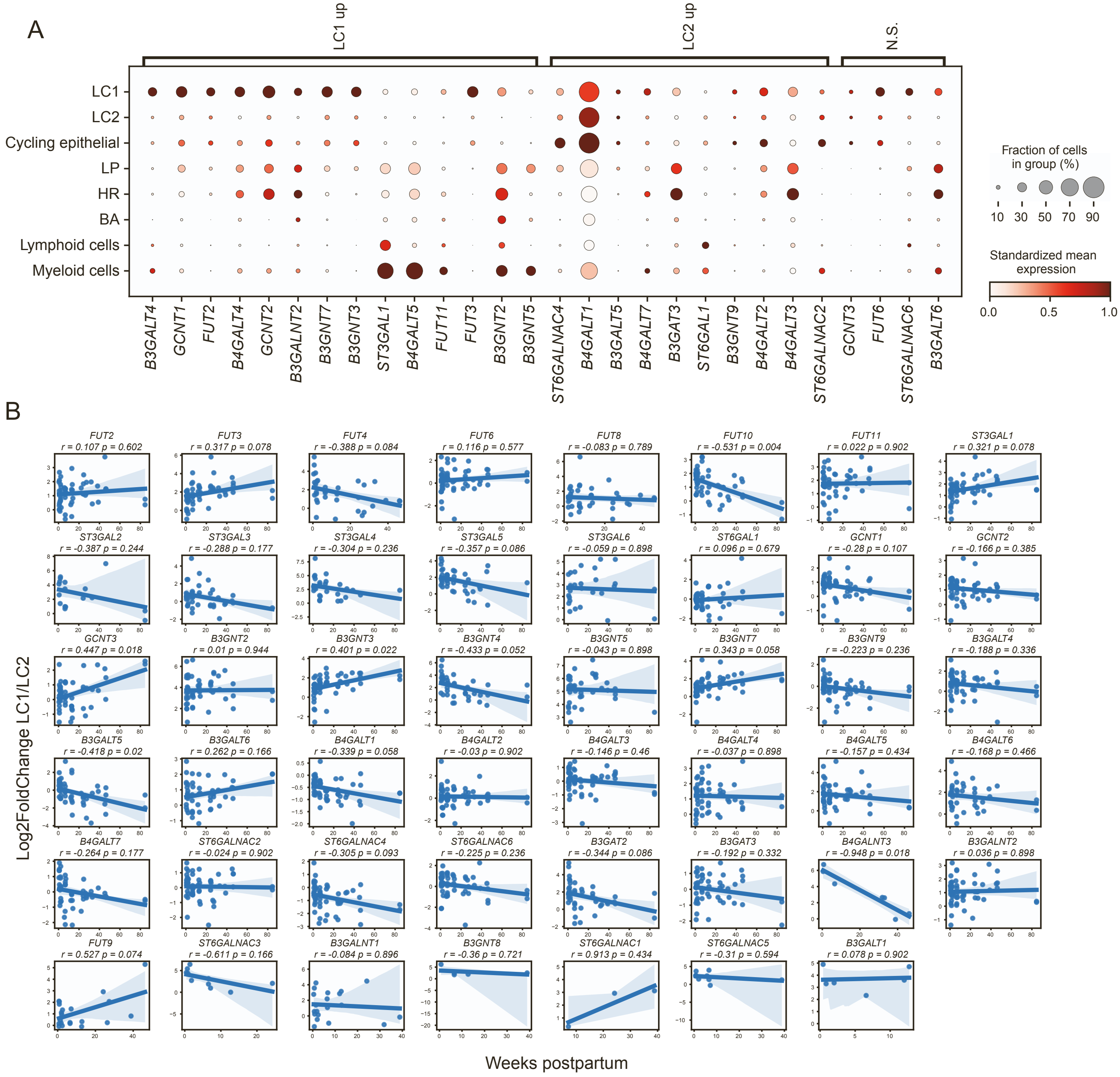

Supplementary Figure 1: **Previously identified HMO synthesis genes have different expression profiles in LC1 and LC2 cells** A. Dotplot showing expression of select candidate HMO synthesis genes (bottom) expressed in a minimum of 10 percent of cells in human milk scRNA-seq data and mammary gland tissue data grouped by celltype (left). LC1 and LC2 enrichment of each gene identified using DESeq2 comparisons of pseudo bulk data between LC1 and LC2 cells in human milk. N.S. genes have large effect size but do not reach significance in the comparison. Dot size indicates percent of cells in group expressing each gene, and dot color indicating column-standardized mean expression of each gene across cell type groups. Mammary gland tissue cell types: LP- luminal progenitor, HR- hormone responsive, BA - basal cell. B. Spearman correlation of the log2FoldChange between expression in LC1 cells and LC2 cells of potential HMO synthesis genes with time postpartum.

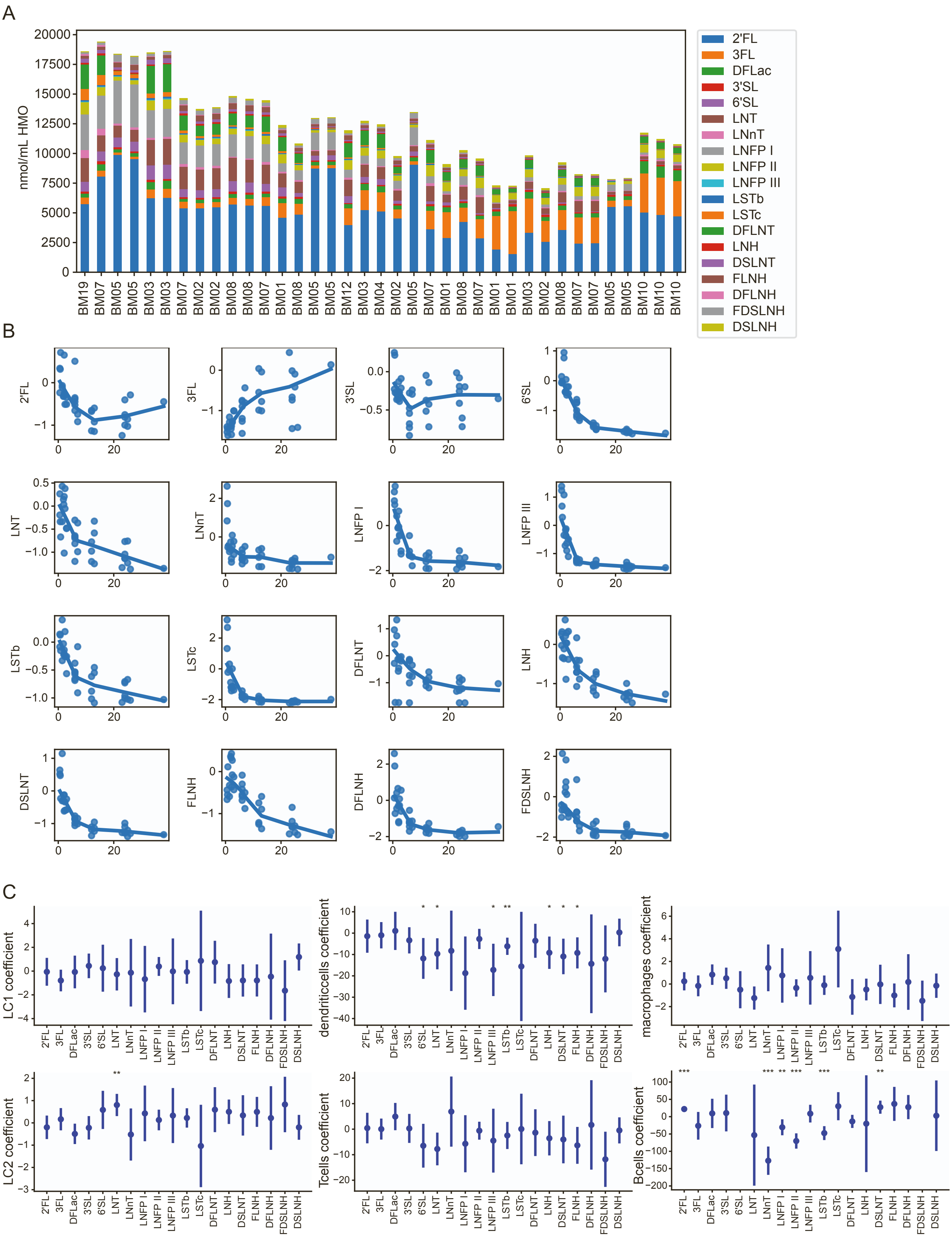
A
nmol/mL HMO
20000
17500
15000
12500
10000
7500
5000
2500
0
BM19 BM07 BM05 BM05 BM03 BM03 BM07 BM02 BM02 BM08 BM08 BM07 BM01 BM08 BM05 BM05 BM12 BM03 BM04 BM02 BM05 BM07 BM01 BM08 BM07 BM01 BM01 BM03 BM02 BM08 BM07 BM07 BM05 BM05 BM10 BM10 BM10
2'FL
3FL
DFLac
3'SL
6'SL
LNT
LNnT
LNFP I
LNFP II
LNFP III
LSTb
LSTc
DFLNT
LNH
DSLNT
FLNH
DFLNH
FDSLNH
DSLNH
B
2'FL
3FL
3'SL
6'SL
LNT
LNnT
LNFP I
LNFP III
LSTb
LSTc
DFLNT
LNH
DSLNT
FLNH
DFLNH
FDSLNH
C
LC1 coefficient
dendriticcells coefficient
macrophages coefficient
LC2 coefficient
Tcells coefficient
Bcells coefficient
2'FL 3FL DFLac 3'SL 6'SL LNT LNnT LNFP I LNFP II LNFP III LSTb LSTc DFLNT LNH DSLNT FLNH DFLNH FDSLNH DSLNH

Supplementary Figure 2: **HMO concentrations and their associations with cell type proportions.** A. Sample composition of HMO concentrations ordered by increasing time postpartum, including samples with matched scRNA-seq data. For samples with multiple replicates, mean HMO concentrations are visualized.
B. As in Fig. 4B, Loess fits for significant association between HMO concentration and weeks postpartum. Scatter plots indicate HMO concentrations by sample. Significance determined by linear mixed model fits $p <= 0.05$.
C. Linear mixed model coefficients for association between HMO concentration and matched immune cell type proportion *$p<=0.05$, **$p<=0.01$,***$p<=0.001$.

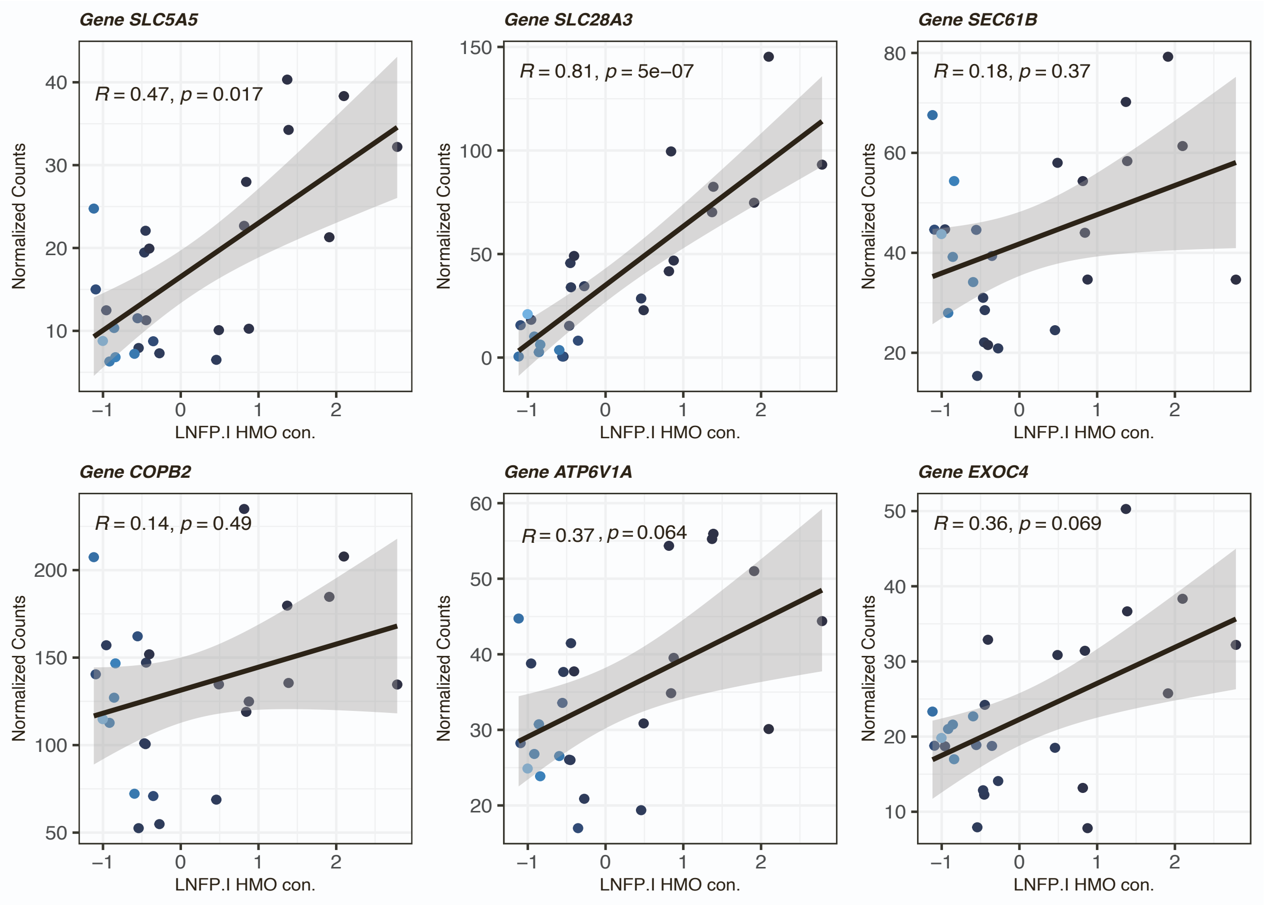

Supplementary Figure 3: **HMO concentrations and their associated gene expression in LC1**: Linear regression between normalized counts of select transporter and enzyme genes (DESeq2 BH adjusted p-values < 0.05) associated with normalized LNFPI concentration in LC1 cells with 95% confidence intervals. (See also table S4)

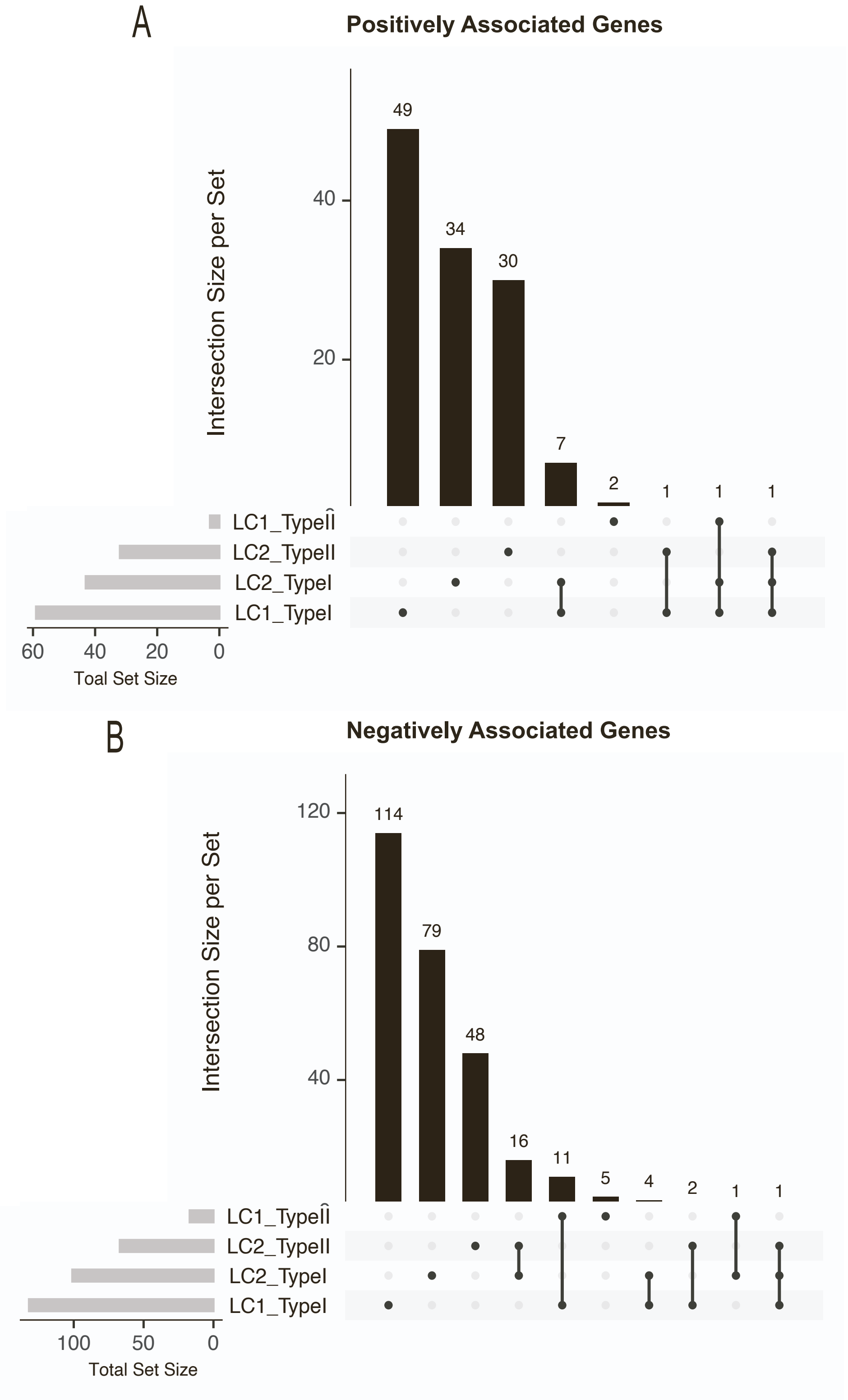
A
Positively Associated Genes
Intersection Size per Set
40
20
49
34
30
7
2
1
1
1
LC1_TypeII
LC2_TypeII
LC2_TypeI
LC1_TypeI
60
40
20
0
Toal Set Size
B
Negatively Associated Genes
Intersection Size per Set
120
80
40
114
79
48
16
11
5
4
2
1
1
LC1_TypeII
LC2_TypeII
LC2_TypeI
LC1_TypeI
100
50
0
Total Set Size

Supplementary Figure 4: UpSet plot showing the number of uniquely associated or intersecting genes (vertical bars) which were associated with Type I and Type II HMO concentration in LC1 and LC2 cell type. A. UpSet plot only for upregulated genes. B. UpSet plot only for downregulated genes (bottom). DESeq2 BH adjusted p-value = <0.05 for all genes. The horizontal bars indicate total number of genes in the set

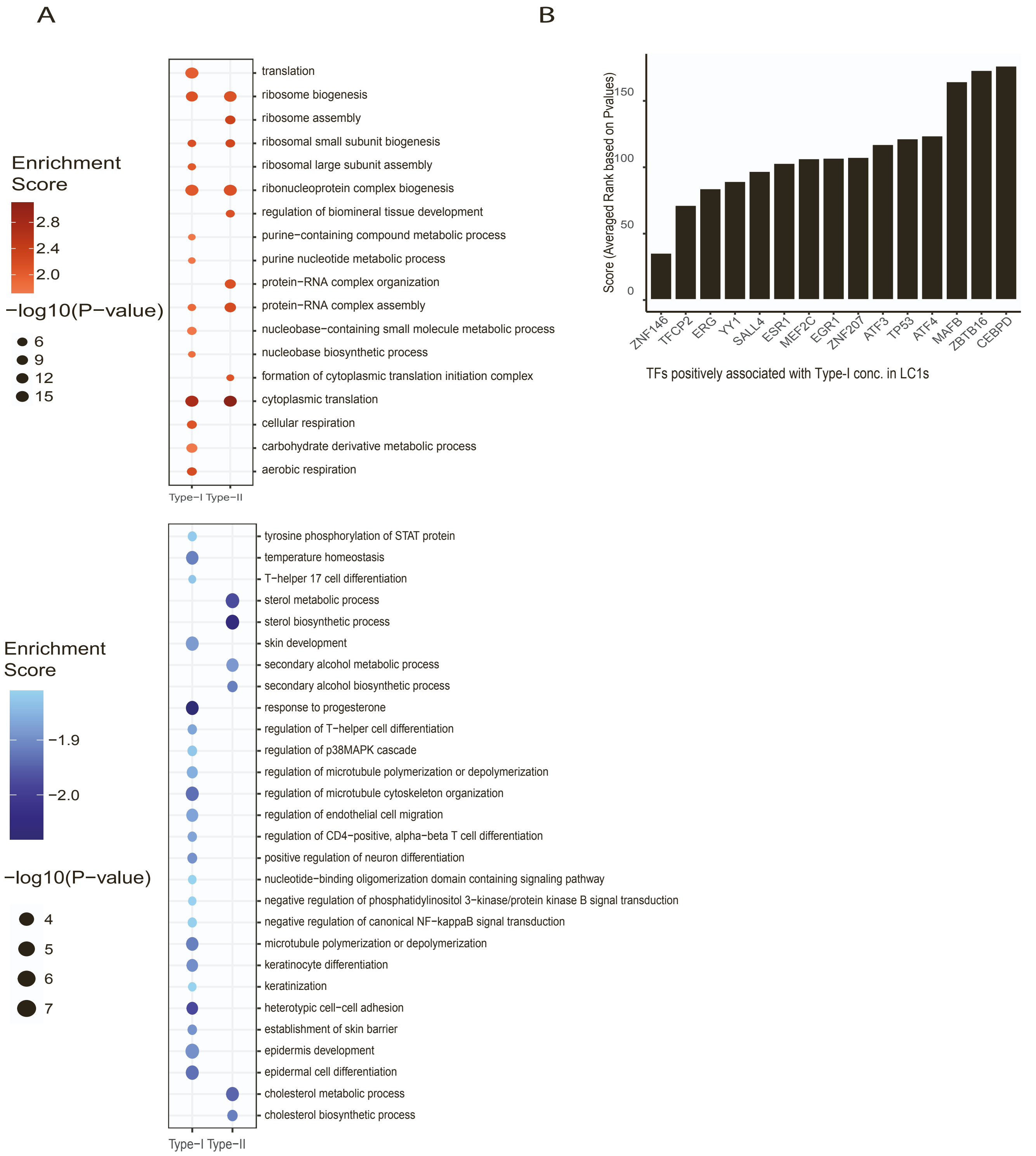

Supplementary Figure 5: **GSEA and TF analysis on HMO concentration-associated genes in LC1**. A. GSEA GO BP dotplot of top positively enriched pathways (top) and top negatively enriched pathways (bottom) associated with Type I or Type II HMO concentrations in LC1. FDR < 0.05 B. Top 15 transcription factors associated with upregulated DEGs associated with Type-I HMO concentration in LC1 from CHEA analysis. The mean scores are averaged ranks of each TF across multiple gene sets in the CHEA database. The mean scores are averaged ranks of each TF across multiple gene sets in the CHEA database. The rank of each TF in a gene set is assigned based on BH adjusted p-values. Lower p-values (higher significance) indicate lower integer ranks and lower overall scores (see methods section).
